# Three-dimensional visualisation of developmental stages of an apicomplexan fish blood parasite in its invertebrate host

**DOI:** 10.1186/1756-3305-4-219

**Published:** 2011-11-22

**Authors:** Polly M Hayes, David F Wertheim, Nico J Smit, Alan M Seddon, Angela J Davies

**Affiliations:** 1 School of Life Sciences, Faculty of Science, Engineering and Computing, Kingston University, Kingston upon Thames, Surrey KT1 2EE, UK; 2 School of Computing and Information Systems, Faculty of Science, Engineering and Computing, Kingston University, Kingston upon Thames, Surrey KT1 2EE, UK; 3 Department of Zoology, The Natural History Museum, London SW 7 5BD, UK; 4 School of Environmental Sciences and Development, North West University, Potchefstroom, South Africa

## Abstract

**Background:**

Although widely used in medicine, the application of three-dimensional (3D) imaging to parasitology appears limited to date. In this study, developmental stages of a marine fish haemogregarine, *Haemogregarina curvata *(Apicomplexa: Adeleorina), were investigated in their leech vector, *Zeylanicobdella arugamensis; *this involved 3D visualisation of brightfield and confocal microscopy images of histological sections through infected leech salivary gland cells.

**Findings:**

3D assessment demonstrated the morphology of the haemogregarine stages, their spatial layout, and their relationship with enlarged host cells showing reduced cellular content. Haemogregarine meronts, located marginally within leech salivary gland cells, had small tail-like connections to the host cell limiting membrane; this parasite-host cell interface was not visible in two-dimensional (2D) light micrographs and no records of a similar connection in apicomplexan development have been traced.

**Conclusions:**

This is likely the first account of the use of 3D visualisation to study developmental stages of an apicomplexan parasite in its invertebrate vector. Elucidation of the extent of development of the haemogregarine within the leech salivary cells, together with the unusual connections between meronts and the host cell membrane, illustrates the future potential of 3D visualisation in parasite-vector biology.

## Findings

**Figure 1:**
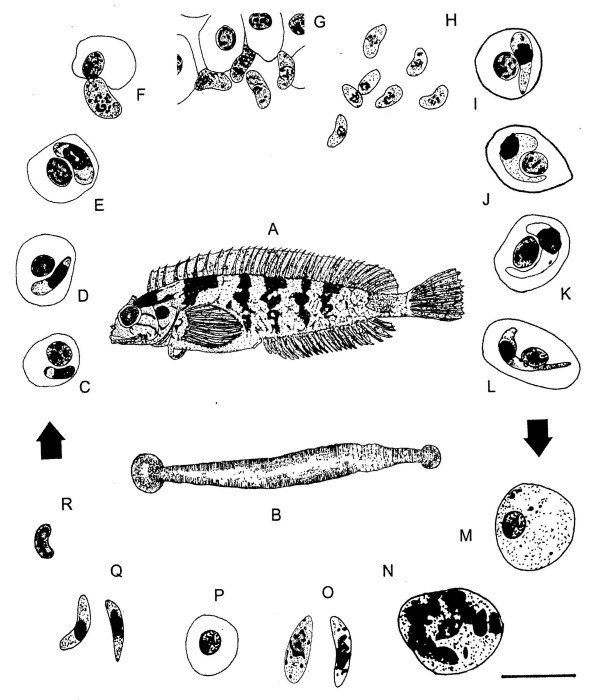
**A-R. Life cycle of *Haemogregarina curvata***. **A**: *Clinus cottoides *redrawn from Penrith [26] (not drawn to scale). **B**: Adult *Zeylanicobdella arugamensis *redrawn from De Silva [27] (not drawn to scale). **C-R**: Microscope drawings to illustrate the life cycle of *Haemogregarina curvata*. **C-L**: Stages within peripheral blood smears from *C. cottoides*. **M-R**: Stages within *Zeylanicobdella arugamensis *squashes and histological sections through salivary glands. Arrows indicate stages of suspected transfer of haemogregarine from one host to another. **C**: Small intraerythrocytic trophozoite. **D**: Larger trophozoite. **E**: Intraerythrocytic meront. **F**: Extracellular meront. **G, H**: Extracellular merozoites. **I**: Intraerythrocytic pregamont form. **J**: Immature gamont. **K **Intermediate gamont. **L**: Mature intraerythrocytic gamont. **M **Immature oocyst. **N **Developing oocyst with 8-10 nuclei. **O**: Free sporozoites. **P**: Meront. **Q**: First generation merozoites. **R**: Second generation merozoite. Scale bar = 10 μm.

Haemogregarines are elongate, protistan parasites found in the blood cells of a number of vertebrate hosts, especially marine fishes [1-4]. Fish haemogregarine life cycles are largely unknown, although some are suspected to involve leech vectors [5-11], while others may utilize crustacean isopods as invertebrate hosts [4,12-14]. Recently, Hayes *et al*. [11] described developmental stages of the marine fish haemogregarine *Haemogregarina *(sensu lato) *curvata *Hayes, Smit, Seddon, Wertheim and Davies, 2006 (Apicomplexa: Adeleorina) from the bluntnose klipfish *Clinus cottoides *(Valenciennes, 1836) and the rhyncobdellid leech *Zeylanicobdella arugamensis *De Silva, 1963. The life cycle for *H. curvata *is shown in Figure 1, including sporozoite, merozoite and meront stages imaged in the current study (Figure 1O-R).

This apicomplexan was only the third valid species of marine fish haemogregarine to have been named from South Africa [11,12,15-18], and the only species from this region for which developmental stages within a leech vector have been described [11]. Stages of *H. curvata *observed by Hayes *et al*. [11] in squash preparations of *Z. arugamensis *included gamonts, oocysts, sporozoites and merozoites. In histological sections of the same leech sporozoites and merozoites were located in tissue adjacent to and within the dorsal sinus, while sporozoites, meronts and merozoites were found in leech salivary gland cells close to the proboscis [11].

**Figure 2:**
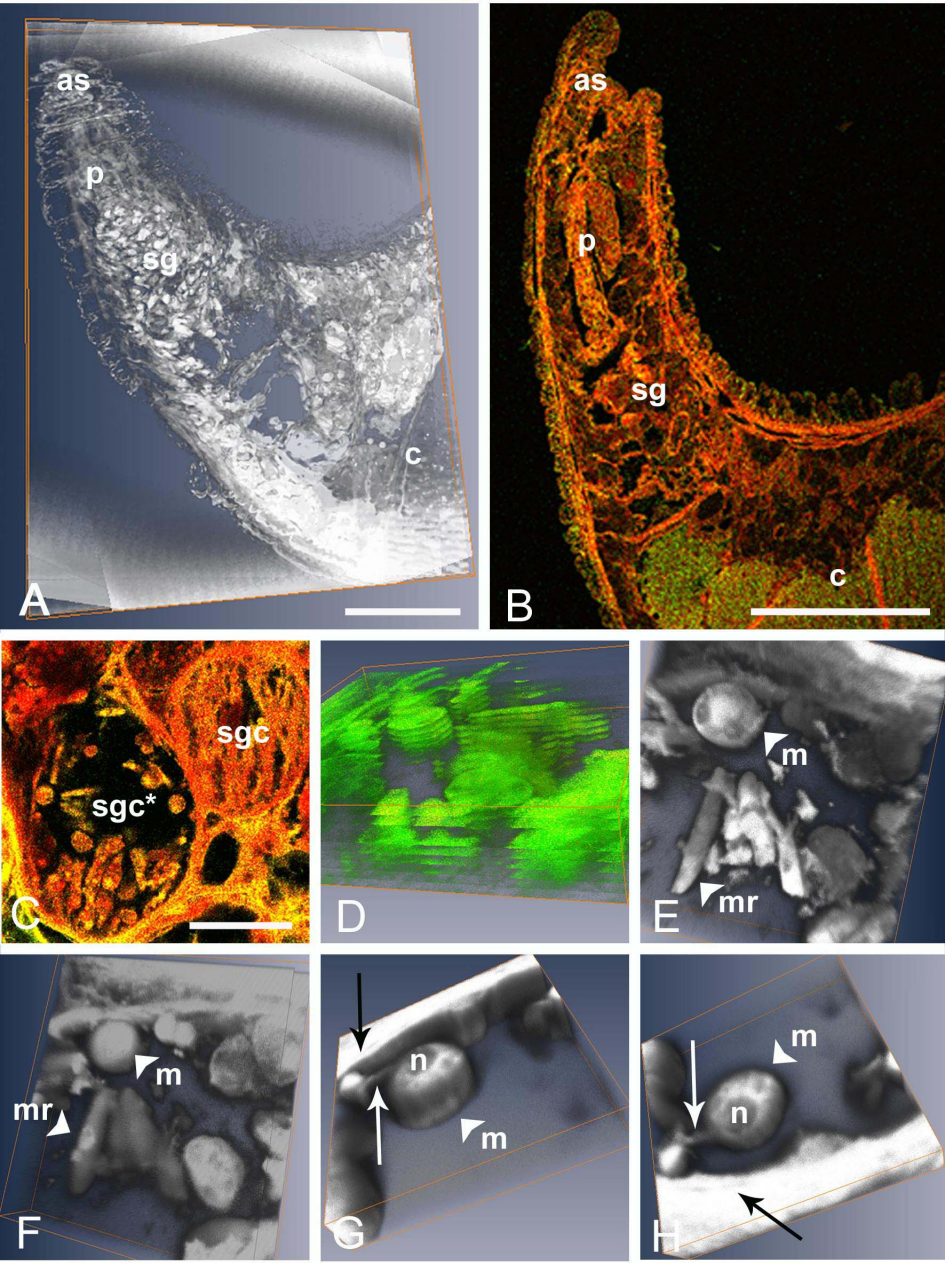
**A-H. Three-dimensional (3D) visualisations (A, D-H) and confocal images (B, C; green and red channel overlays) of the leech *Zeylanicobdella arugamensis *and developmental stages of *Haemogregarina curvata *within its salivary gland cells**. **A**: Voltex 3D visualisation of bright field microscopy images of stacked haematoxylin and eosin (H&E) stained histological sections (n = 10), and **B**: a confocal image of a single H&E stained section, through the anterior end of *Z. arugamensis*, showing anterior sucker (as), proboscis (p), salivary glands (sg) and crop (c). Scale bars: A and B, 0.2 mm, Z axis in A not to scale. **C**: Non-infected (sgc) and infected (sgc*) leech salivary gland cells. Scale bar = 20 μm. **D - F**: Stacked orthoslice (D) and voltex (E-H) 3D visualisations of 10 stacked confocal images of infected salivary gland cells, showing haemogregarine stages at various angles of rotation (green and red channels displayed), with meront (m) and merozoite (mr) stages. **G-H**: 3D visualisation of 26 stacked confocal images of a marginally located meront (m) with prominent nucleus (n) and a tail-like connection (large arrows) to the salivary gland cell limiting membranes at different rotations. X-Y-Z axis dimensions for visualisations = 30 μm × 30 μm × 6 μm (D-F), 17 μm × 17 μm × 3 μm (G-H).

Subsequent to the published description of *H. curvata*, it became possible to examine the developmental stages of this parasite further. This was achieved using histological sections of infected leech salivary glands and by investigating the parasite-host cell relationship by three-dimensional (3D) visualisation, linked to brightfield or confocal microscopy. This revealed new insights into the development and parasite-host cell interactions of the apicomplexan within its leech vector (Figure 2A-H).

3D images of the stages of *H. curvata *within the salivary gland cells of *Z. arugamensis *were achieved by the visualisation and modelling software Amira 4.0 (Mercury Group Systems, USA). This system was used to create 3D visualisations of two-dimensional (2D) digital brightfield microscopy images (a stacked series of histological sections) and confocal microscopy images (a stacked series of scans through individual histological sections). Images of haematoxylin and eosin stained histological sections were captured as detailed by Hayes *et al*. [11]. Prior to 3D visualisation, brightfield images were aligned, manually if necessary, using the Amira software. As confocal images were a series of scans through single histological sections (typically, z stacks of 10-26 slices), additional alignment was not necessary.

In Amira 4.0, following alignment of brightfield microscopy images, the brightness and contrast were adjusted, and a median filter (5 × 5 kernel) was applied to reduce noise before further brightness and contrast adjustments were made. The images were then stacked and displayed as 3D visualisations of individual orthoslices, or using a 3D volume rendering (voltex) module which displayed the images as a texturised 3D visualisation. Confocal images applied directly to the 3D imaging software, were also viewed as either individual orthoslices, or using the voltex module (both green and red channels were displayed showing autofluorescence from the sample sections using the argon (488 nm excitation) and helium/neon (453 nm excitation) lasers, and XYZ coordinates set, as appropriate).

Stacked brightfield images of histological sections (n = 10) through the anterior portion of *Z. arugamensis *displayed either as individual orthoslices or as volume rendered (voltex) visualisations, provided a good overview of this region of the internal anatomy of the leech, including the position, form and spatial layout of the salivary glands and proboscis (Figure 2A). Recently, Davies *et al*. [4] reported using similar methods to investigate the internal anatomy of juvenile gnathiid isopods and the potential use of the techniques in locating the development sites of another fish haemogregarine, *Haemogregarina bigemina *Laveran and Mesnil, 1901 [4,12,13,15-17].

3D visualisations of confocal images of the salivary gland cells of *Z. arugamensis *infected with *H. curvata *were superior to stacked brightfield images of this region in quality, providing an appreciation of the morphology and arrangement of the developmental stages of the haemogregarine, and their relationship with the host cells (Figure 2B-H). Furthermore, volume rendered (voltex) 3D images (Figure 2E-H), in contrast to those displayed as individual orthoslices (Figure 2D), appeared to provide the clearer delineation of structure.

Stacked orthoslice and voltex visualisations of 10 confocal images through single histological sections of salivary gland cells showed merozoites, sporozoites and meronts in some detail. These stages lay freely within enlarged salivary gland cells as individuals, or in small clusters, especially marginally, adjacent to the salivary cell limiting membrane (Figure 2E-H). Voltex displays, in particular, showed the surface morphology of individual stages and their spatial layout (Figure 2E-H). These images also illustrated the reduced cellular contents of infected host cells reported by *Hayes et al*. [11] with host cell content remnants visible among the haemogregarine stages (Figure 2E-H); this suggests a pathological effect of *H. curvata *infection on its invertebrate host, as indicated by Hayes *et al*. [11]. When examined closely using voltex displays of 10-26 confocal images, the majority of meronts located marginally within salivary gland cells were seen with small tail-like connections to the host cell limiting membrane (Figure 2G-H). This host cell-parasite interface had not been visible in 2D light micrographs [11]. The purpose of this tail-like structure is not clear; it may be essential for the continued growth and development of the meront stage within the salivary cell, acting as a conduit for nutritional needs, or perhaps it is a remnant of earlier development (of sporozoites or a previous generation of merozoites) within the host cell. Uni *et al*. [19] observed, by transmission electron microscopy, a connection between the coccidian *Cryptosporidium muris *(Strain RN66) and mouse gut mucus cells in the form of an annular ring; this structure was interpreted as having a role in anchoring the parasite to the inner region of the host cell.

Despite being used widely in medicine, for example, in displaying Magnetic Resonance Images [20-22], the use of 3D visualisation in parasitology, particularly using brightfield and confocal images, appears limited to date [see [23]]. Shinn et al. [24] used 3D reconstruction of scanning electron micrographs to observe structural details of the marginal hooks of monogenean parasites of fishes, while Ligasová et al. [25] produced a 3D model of secretory glands in cercaria of the neuropathogenic schistosome *Trichobilharzia regenti *using confocal microscopy. To the authors' knowledge this is the first account of the use of 3D visualisation to examine the developmental stages of an apicomplexan parasite in its invertebrate host. The ability to reveal the extent of development of the haemogregarine within leech salivary cells, together with the unusual connections between meronts and the host cell membrane, illustrates the future potential of this technique in this field.

## Competing interests

The authors declare that they have no competing interests.

## Authors' contributions

PMH conceived the project, carried out the experimental work, participated in the data analysis and wrote the manuscript. DFW conceived the project, participated in the data analysis and provided significant support to the preparation of the manuscript. NJS organised sample collection and, with AMS, participated in drafting the manuscript. AJD conceived the project and provided significant support to the preparation of the manuscript. All authors read and approved the final manuscript.
